# Tracking breastfeeding and weaning practices in ancient populations by combining carbon, nitrogen and oxygen stable isotopes from multiple non-adult tissues

**DOI:** 10.1371/journal.pone.0262435

**Published:** 2022-02-02

**Authors:** Yadira Chinique de Armas, Anna-Maria Mavridou, Jorge Garcell Domínguez, Kaitlyn Hanson, Jason Laffoon

**Affiliations:** 1 Department of Anthropology, The University of Winnipeg, Manitoba, Canada; 2 Faculty of Archaeology, Leiden University, Leiden, The Netherlands; 3 Consejo Nacional de Patrimonio Cultural, La Habana, Cuba; University at Buffalo - The State University of New York, UNITED STATES

## Abstract

This paper explores the potential of combining different isotope systems from different tissues to improve resolution when reconstructing breastfeeding and weaning practices (BWP) in archaeology. Additionally, we tested whether changes in diet can be detected in deciduous teeth. Rib collagen samples from 22 infants/children from the archaeological site of Bacuranao I (Mayabeque, Cuba) were processed for nitrogen (δ^15^N) and carbon (δ^13^C_co_) stable isotopes and assessed using a Bayesian model (WARN). In addition, enamel of 48 teeth from 30 infants/children were analyzed for oxygen (δ^18^O_en_) and carbon (δ^13^C_en_) stable isotopes. Data revealed that the timing of weaning cannot be characterized precisely by analyzing either δ^18^O or δ^15^N. While a depletion in both δ^15^N and δ^13^C_co_ is only evident after one year, the WARN model suggested that the weaning process started at around 3 months and ended around 1.7 years. Most teeth were enriched in δ^18^O_en_ compared to deciduous incisors, suggesting a breastfeeding signal. However, a high variability in δ^18^O was found between similar teeth from the same individuals. Higher enrichment in δ^18^O_en_, and variability, was observed in tissues formed during the first six months of life. A δ^13^C enrichment of 1.0‰ was observed among deciduous teeth and ribs. While most individuals enriched in δ^15^N showed enrichment in δ^13^C, the δ^18^O values were more variable. Our data suggests that stable isotopes of deciduous teeth, especially δ^13^C_en_, can be used to detect changes in diet during the weaning process. It is also possible that the δ^18^O enrichment observed in M1 is influenced by the effects of cooking techniques on weaning foods. The combination of multiple isotope systems and tissues overcome some of the limitations posed by single tissue approaches.

## Introduction

Refining models to reconstruct breastfeeding and weaning practices (BWPs) of past populations is of great interest to bioanthropologists, as it provides information about demographic patterns, health [1–5], non-adult rearing practices [6, 7], and subsistence strategies [8–10]. In relation to paleodemography, higher fertility rates and reduced births intervals have been associated with shorter breastfeeding stages [11–13]. Breast milk is critical for the survival of infants since it provides them with a suitable high-energy diet for their rapid growth and development [14, 15], and contributes to passive immunity to protect against the harmful effects of various pathogens [16–19]. Studies on modern populations suggest that after six months of age, infant nutritional needs require the incorporation of more complex foods [20, 21], which marks the beginning of the weaning process. The type of food used during the weaning process varies considerably between populations and is influenced by dietary traditions, the availability of resources, infant preferences, and their sex and health status [22, 23]. The length of the weaning process is also variable among groups, which ends with the complete cessation of breastfeeding [2, 24].

Most BWPs studies of ancient populations have analyzed nitrogen stable isotope values in bone collagen (δ^15^N), as its isotopic composition enriches progressively through trophic levels [25–27]. Given that breast milk is the only source of nutrients of a nursing infant, the δ^15^N value of bone collagen increases by ~2–3‰ for exclusively breastfed human infants after birth [28–31]. Subsequently, the incorporation of supplements into the infant’s diet is signalled by a gradual decrease in nitrogen isotope values [32]. In several studies, nitrogen isotope values have been combined with collagen carbon isotope values (δ^13^C_co_) [30, 31, 33–35], providing further insights into the types of foods used during supplementation. A few studies have alternatively used δ^18^O values to reconstruct BWPs in ancient populations [36–39]. This is based on the principle that bioapatite δ^18^O records the oxygen isotope chemistry of drinking water sources in all but nursing infants, whose δ^18^O values are enriched by the intake of breast milk formed from body water, which itself has been enriched by maternal expiration of isotopically depleted water vapour [40, 41]. Because of this effect, tissues formed before the end of weaning have been observed to be isotopically enriched between 0.5% and 2‰ in comparison to similar maternal tissues [36, 37, 42].

Recent studies of BWPs in Caribbean populations combined the analysis of δ^15^N and δ^13^C values in bone collagen with the use of Bayesian models to reconstruct the timing of weaning and the type of foods used during their weaning process [8, 9]. The age at the start of weaning was found to be significantly later than observed in ethnographic populations from the area [43–45], or the age established by modern medical recommendations [46], an issue that had previously been observed when analyzing archaeological metadata more broadly [24]. A delayed signal of supplementation in nitrogen isotope values is likely associated to the fact that δ^15^N values reflect the protein source of diet [47], while many typical weaning foods are high in carbohydrates and low in protein [45, 46], and thus nitrogen isotopes are rather insensitive proxies for detecting the start of the weaning process. This is further influenced by the fact that changes in δ^15^N are not reflected instantly in bones due to bone collagen turnover rates [24]. Because these early weaning foods would require the introduction of external water sources to infant diets, δ^18^O values from an individual’s tissues theoretically have the potential to detect the start of weaning with greater precision than δ^15^N values. However, δ^18^O values can vary substantially due to local, and regional, geographic and climatic conditions, including temperature variations, geographic patterns in precipitation, and seasonality [48–52]. Consequently, the scale of δ^18^O changes reflecting breastfeeding may be small compared to other natural sources of isotopic variation.

In this paper, we analyzed δ^15^N and δ^13^C_co_ in bone collagen, and δ^13^C_en_ and δ^18^O values of tooth enamel from the archaeological site of Bacuranao I (Mayabeque, Cuba) to evaluate the potential of combining different isotope systems to gain better resolution in the reconstruction of BWPs in archaeological populations. The large number of well-preserved deciduous and permanent teeth from infants/children at Bacuranao I provided a unique opportunity to explore to what extent changes in dietary practices can be detected in tissues formed at early ages, a perspective recently shown to be promising when comparing a few second deciduous molars with teeth formed at later life stages [39]. Combining different stable isotope elements of tissues formed at different ages (deciduous, permanent molars and ribs) offers potential for improving the isotopic methods used to study BWPs by refining the resolution at which the timing of weaning can be detected in ancient populations.

### Isotopic approaches to breastfeeding and weaning practices in archaeology

**Fig 1:**
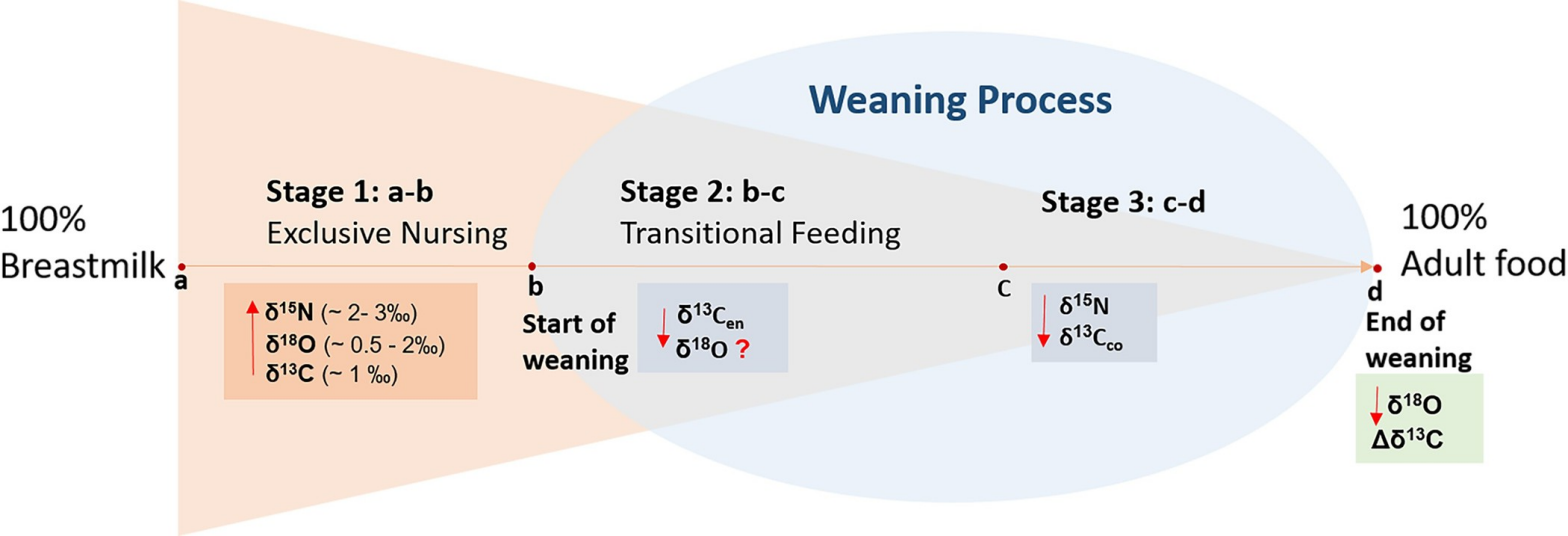
Stable isotopes during the different stages of the weaning process.

In this paper, we refer to weaning as a process that starts when foods other than breast milk are continuously included in the diet, and ends with the cessation of breastfeeding [2, 24, 46]. Human breastfeeding and weaning practices (BWPs) have been modeled into four stages or periods: 1) exclusive breastfeeding (diet consists entirely of breastmilk); 2) diet consisting of a combination of breastmilk and complementary foods (nutritionally rich and relatively sterile foods used to feed infants and toddlers); 3) breastfeeding plus complementary and adult foods (foods typically used by the juveniles and adults of the population) and; 4) exclusive consumption of adult foods [53] (Fig 1). During the weaning period (stages 2 and 3), breastmilk still remains an important source of nutrients and immunity to the infant’s body systems, with complementary and adult foods increasingly contributing to infant’s total food intake while the frequency of suckling diminishes gradually until the end of the weaning process. The gradual transition from 100% breastmilk to 100% adult food is a complex and variable process that offer multiple challenges when attempting to identify the stages of BWPs in skeletal tissues from their stable isotope values, especially in non-adults.

The organic or the inorganic part of bones and teeth can be sampled for stable isotope analysis to gain insights on various aspects of BWPs of past populations [2, 28, 30, 36, 37]. The choice of the sampling material has a direct link with the kind of questions that can be answered. The organic phase of skeletal tissues (bone and dentin collagen) primarily reflects the protein component of diet, with ~74 ± 4% of collagen estimated to derive directly from consumed protein sources [47]. By contrast, the inorganic phase of skeletal tissues (bone apatite and enamel) reflects an average of whole diet including all three macronutrients: carbohydrates, lipids, and protein [54]. Many early isotopic approaches to the study of BWPs focused primarily, or exclusively, on carbon and/or nitrogen isotope analyses of bone collagen from individuals that died in infancy or early childhood [38]. The phenomenon of bone turnover can influence the nature of the information that can be derived from those isotopic analyses and the period of life reflected in the isotope data [24, 55]. The choice of skeletal element sampled, from slower growing components such as femur to bones with more rapid turnover rates such as ribs impacts the time period represented in the stable isotope values [24].

Dental samples can be also highly informative for BWP studies. Teeth develop during the earliest period of an individual’s life, from *in utero* to late childhood depending on the tooth type, and the patterns of their growth have been thoroughly studied and defined [56, 57]. Teeth in general are not subjected to turnover and remodeling [58], therefore the chemical composition of dental tissues reflect specific and relatively short periods of an individual’s life. Hence, teeth can be used as a valuable source of evidence and give insights into dietary changes during specific periods of infancy and childhood [30, 36, 37, 39, 59].

A few isotope studies of BWPs have focused on carbon and oxygen isotope analysis of bioapatite (biological hydroxyapatite) in dental enamel. As bioapatite is formed from dissolved bicarbonates in blood water, it is assumed that the bioapatite in dental enamel should reflect dietary consumption during the period of enamel formation/mineralization [58, 60]. In this regard, it may not suffer as much from the temporal delay associated with protein biosynthesis that may complicate BWP studies which rely exclusively on bone collagen. However, enamel consists almost exclusively of bioapatite and contains no measurable collagen, and therefore nitrogen isotope analysis cannot be conducted on dental enamel. Carbon isotope analysis of dental enamel may, in some cases, be well suited for identifying the introduction of supplemental foods to diet (the onset of weaning), especially in cases where supplemental foods are low in protein and thus less likely to be detected in collagen carbon and nitrogen isotope signals. Oxygen isotope analysis of enamel is generally considered to be reflective of consumed water. For breastfeeding infants and children, consumed water derives exclusively from breastmilk, which has been demonstrated to be slightly enriched in ^18^O relative to maternal δ^18^O. As such, oxygen isotope analysis of enamel from different tissues (teeth) formed at different periods offers the potential for identifying the introduction of complementary food and drinks. Nevertheless, the interpretation of oxygen isotope data is highly complex owing to the numerous sources of variation, and the presumed enrichment of ^18^O in tissues formed during the breastfeeding period has not yet been systematically tested on populations from diverse geographic and cultural contexts.

Most studies that have used stable isotope analysis of enamel to detect the timing of weaning relied on differences between the first permanent molars (M1, formed during infancy) and teeth whose formation completes after infancy such as canines, premolars and third permanent molars [36, 37, 39, 42]. The main limitation of such approaches is that the first molar enamel forms from roughly birth to 2.5–3 years of age. As such, for most if not all individuals, this will incorporate tissue formed during both the (early) exclusive breastfeeding period and the (later) weaning period. Such sampling strategies make it difficult to interpret enamel isotope results in reference to the main periods of BWPs, and fail to capture tissues that form exclusively, or even primarily, in the earliest breastfeeding period. One way to overcome this complication is to modify sampling strategies to incorporate deciduous teeth that form earlier in life and therefore reflect diet in the pre-weaning period (both *in utero* and during the early exclusive breastfeeding period). In other words, studies using multiple dental elements sampled from the same individual to investigate BWPs may benefit from the inclusion of deciduous teeth that better reflect the pre-weaning period. More specifically, the enamel of certain deciduous teeth primarily form *in utero* and thus provide a baseline for pre-birth isotope values (in principle in equilibrium with maternal isotope values), whereas enamel from other deciduous teeth form in the first few months after birth and thus should better reflect the exclusive breastfeeding stage of early infancy. This comparative approach can potentially provide insights into the timing of important transitions within the weaning trajectory but are hampered by temporal delays between consumption and incorporation into skeletal tissues as well as the osteological paradox [61].

Breastfeeding and weaning practices are highly related to the underlying pathology of the non-adult individuals since the isotopic signatures will show signals of ill children who did not survive into adulthood. Although the effects of all diseases have not been studied yet, the variation of the δ^18^O signatures due to metabolic diseases has been examined [42]. Therefore, biases can be introduced in the study due to the representation of exclusively dead individuals. In this way the sample does not reflect the objective heterogeneity of the population and any reconstruction which is created will not be an equal reflection of the living population [61]. Furthermore, the incorporation of samples from individuals that suffered but survived may also hide biases due to potential alterations in their isotopic chemistry caused by the disease [62]. At the same time, sampling of individuals who died at a young age may skew patterns of breastfeeding observed in the broader population, as these individuals might have differed in the nutrition they received. Other broader osteological issues, such as the natural variability in infant growth that leads to uncertainties in ageing infant remains (sometimes by as much as half a year or more) and variation in the rhythm of the development of the teeth could influence the level of temporal precision possible in any stable isotope study of non-adults. For this study, we have applied a novel approach combining multiple isotope analyses of specifically targeted multiple skeletal elements including dental enamel of *both* deciduous and permanent teeth, as well as bone collagen, and have assessed the data with a Bayesian mixing model (WARN) and statistical analysis in order test whether such an approach can provide more detailed and robust individual reconstructions of BWPs in archaeological populations.

### Archaeological context

**Fig 2:**
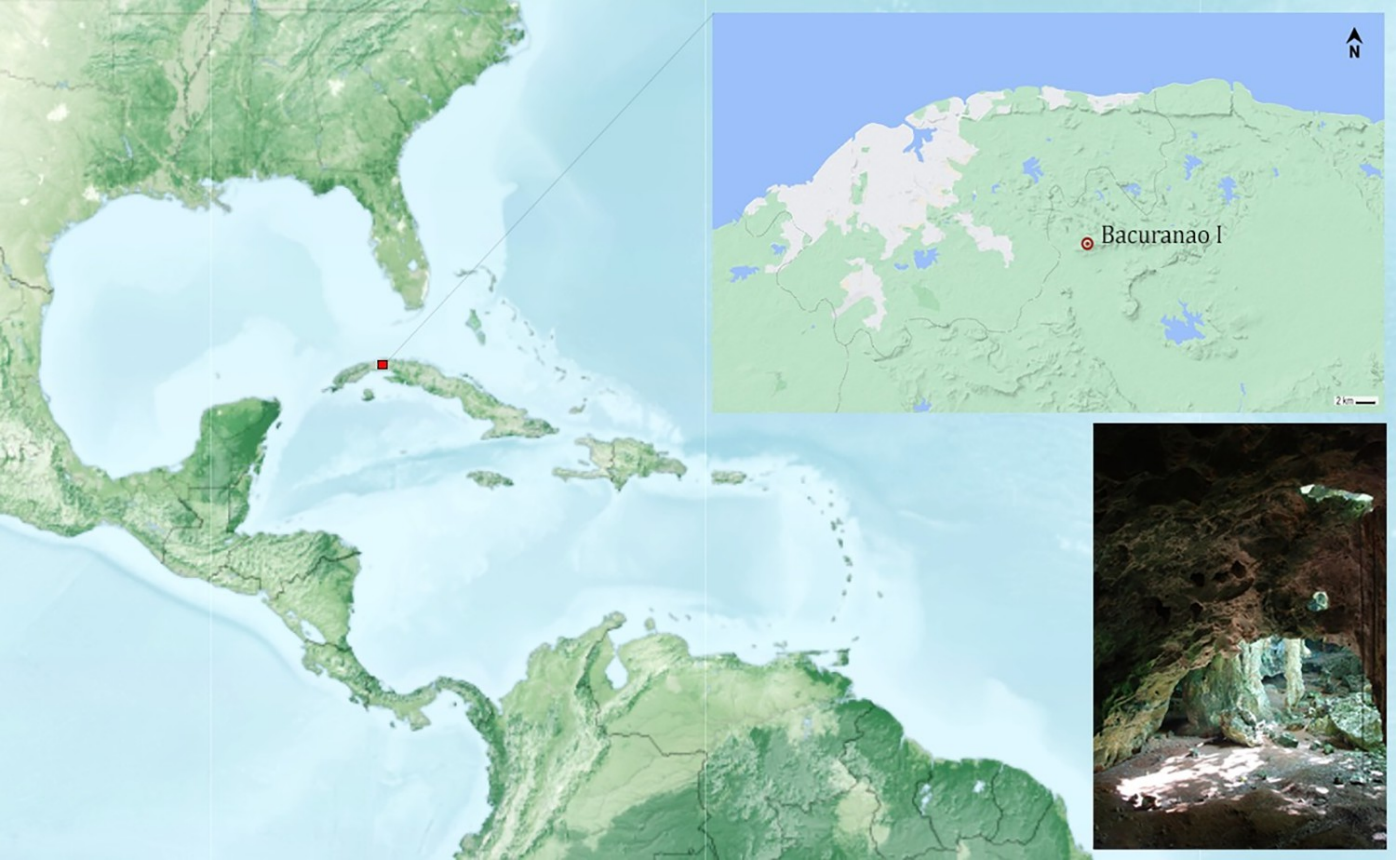
Location of the archaeological site of Bacuranao I, Mayabeque, Cuba.

The archaeological site of Bacuranao I, or Cueva del Infierno, is a cave site of approximately 1044 m^2^ located 15 km inland from the north coast, in the municipality of San Jose de las Lajas, province of Mayabeque in Cuba (Fig 2). The site was classified as ‘Preagroalfarero’ (preagroceramist) [63], or ‘Appropriator’ [64] whose major indicators are equivalent to what it is understood as ‘Archaic Age’ in Antillean archaeology.

Two burial areas, Cemetery I and II, were found in two opposite ends of the cave central sinkhole [65]. Most individuals were found in Cemetery I, including a female adult and 53 non-adults. Cemetery II occupied an area of 5 m^2^ in which the remains of four individuals, comprising adults and non-adults, were placed. Later anthropological studies confirmed a minimum number of 66 individuals (MNI): nine adults and 57 non-adults [66]. From them, 52 individuals were in the category of infants (0–3 years), 75% of which were likely in their first six months of life. In addition, the analysis of 1616 isolated permanent teeth increased the MNI to 169 individuals [65]. The lack of chronological data make it difficult to know if these individuals belong to the same cohort populations, so we acknowledge the potential limitations of the use of the term ‘population’. All necessary permits were obtained for the described study, which complied with all relevant regulations. Permits to study the sample were granted to Jorge Garcell from the Cuban National Heritage Commission. Human remains excavated at Bacuranao I are safeguarded in the Municipal Museum of San Jose in Mayabeque province, Cuba.

## Materials and methods

### The sample

Rib samples from 22 individuals were processed for bone collagen nitrogen (δ^15^N) and carbon (δ^13^C_co_) stable isotope analysis (Table 1). We selected ribs because they have higher turnover rates, which make them a good candidate to reflect the isotopic signals of the individuals at, or near, the time of death. Additionally, we analyzed the enamel of 48 teeth from 30 individuals for oxygen (δ^18^O_en_) and carbon (δ^13^C_en_) [5 deciduous incisors (di); 3 deciduous canine (dc); 6 deciduous first molars (dm1); 16 deciduous second molars (dm2); 17 permanent first molars (M1); 1 permanent canine (C)]_._ Tooth enamel not only represents discrete periods of formation but is also considered to be less prone to diagenesis than the bioapatite fraction of bone [67, 68].

**Table 1:** General information of the Bacuranao I (Mayabeque, Cuba) individuals included in this study.

ID	Context	Estimated Age at Death/Range	Tissue/Crown formation stage^1^	Crown Formation Period^2^	Age at Crown Formation^3^
AdultoB-E-1	C-3, 1.0 m, saco 79	Adult	C/(Cr_c_)	4-5m - 6y	4-5m - 6y
**CI_E-1**	C-3, 1.0 m, saco 79	3 y ± 12 m	dm1/ (Cr_c_)	14.5-17w - 6m	14.5-17w - 6m
			dm2/(Cr_c_)	16–23.5w - 11m	16–23.5w - 11m
**CI_E-1C**	C-3, 1.0 m, saco 79	4 y ± 8 m	dc/(Cr_c_)	15-18w - 9m	15-18w - 9m
			M1/(Cr_c_)	30w - 3y	30w - 3y
**CI_E-1D**	C-3, 1.0 m, saco 79	1 y ± 4 m	dm2/(Cr_c_)	16–23.5w - 11m	16–23.5w - 11m
**CI_E-6A**	C-3, 1.98–1.95 m, saco 168	6 m ± 3m	di/(Cr_c_)	13-16w - 2.5m	13-16w - 2.5m
			dm2/(Cr_1/2_)	16–23.5w - 11m	16–23.5w - 6m
			M1/(Cr_co_)	30w - 3y	30w – 6m
**CI_E-8A**	C-4, 0.87 m, saco 62	0–0.5 y	Ribs	-	-
**CI_E-12A**	C-1, 0.6 m, Saco 49	Adult	Ribs	-	-
**CI_E-18A**	C-4, 1.48–1.70, saco 31	0–0.5 y	Ribs	-	-
**CI_E-19A**	C-4, 1.58–1.79, saco 71	0–0.5 y	Ribs	-	-
**CI_E-21A** *	C-4, 2.44–3.15, saco 69	2 y ± 8 m	di/(Cr_c_)	13-16w - 2.5m	13-16w - 2.5m
			M1/(Cr_3/4_)	30w - 3y	30w – 2y
			Ribs	-	-
**CI_E-21B**	C-4, 2.44–3.15, saco 69	6 m ± 3 m	dm2/(Cr_1/2_)	16–23.5w – 11m	16–23.5w – 6m
**CI_E-21C**	C-4, 2.44–3.15, saco 69	1 y ± 4 m	dc/(Cr_c_)	15-18w – 9m	15-18w – 9m
**CI_E-21D**	C-4, 2.44–3.15, saco 69	3 y ± 12 m	dm2/(Cr_c_)	16–23.5w – 11m	16–23.5w – 11m
**CI_E-21E**	C-4, 2.44–3.15, saco 69	6 y ± 24 m	M1/(Cr_c_)	30w – 3y	30w – 3y
**CI_E-21F**	C-4, 2.44–3.15, saco 69	3–7 y	M1/(Cr_c_)	30w – 3y	30w – 3y
**CI_E-22A**	C-3, 1.87–2.51, saco 101	0–0.5 y	Ribs	-	-
**CI_E-23A**	C-4, 1.80–1.98, saco 34	0–0.5 y	Ribs	-	-
**CI_E-25A**	C-3, 4, D-3, 4, 2.23–2.51, saco 46	0–0.5 y	Ribs	-	-
**CI_E-27A**	C-3, 1.23, saco 76	1.5–2.5 y	Ribs	-	-
**CI_E-28A** *	C-4, 1.68–2.28, saco 48	6 m ± 3 m	dm2/(C_1/2_)	16–23.5w - 11m	16–23.5w - 6m
			Ribs	-	-
**CI_E-29**	A-3, 3.0–3.7, saco 38	9 m ± 3 m / 1y± 4 m	dm2/(Cr_c_)	16–23.5w - 11m	16–23.5w - 11m
**CI_E-29A** *	A-3, 3.0–3.7, saco 38	6 m ± 3 m	dc/(C_3/4_)	15-18w - 9m	15-18w - 6m
			dm2/(C_1/2_)	16–23.5w - 11m	16–23.5w - 6m
			Ribs	-	-
**CI_E-30A** *	A-3, 1.36–2.22, saco 36	6 m ± 3 m	di/(Cr_c_)	13-16w - 2.5m	13-16w - 2.5m
			dm2/(C_1/2_)	16–23.5w - 11m	16–23.5w - 6m
			Ribs	-	-
**CI_E-30**	A-3, 1.36–2.22, saco 36	1y ± 4m	dm2/(Cr_c_)	16–23.5w - 11m	16–23.5w - 11m
**CI_E-31A** *	B-4, 2.0–2.20, saco 54	6 m ± 3 m	dm1/(Cr_c_)	14.5-17w - 6m	14.5-17w - 6m
			Ribs	-	-
**CI_E-33A**	B-1,C-1, 2.23–2.79, saco 39	0–0.5 y	Ribs	-	-
**CI_E-34A** *	B-5, 2.0–2.80, saco 28	Birth ± 2m / 6m ± 3m (~ 3m)	dm1 (C_3/4_)	14.5-17w - 6m	14.5-17w - 3m
			Ribs	-	-
**CI_E-35A**	C-6,C-5,1.60–1.80, saco 166	1 y ± 4 m	di/(Cr_c_)	13-16w - 2.5m	13-16w - 2.5m
			2 dm2/(Cr_c_)	16–23.5w - 11m	16–23.5w - 11m
			Ribs	-	-
**CI_E-36A**	B-5, 1.80, saco 103	18 m ± 6m	dm1/(Cr_c_)	14.5-17w - 6m	14.5-17w - 6m
			dm2/(Cr_c_)	16–23.5w - 11m	16–23.5w - 11m
			Ribs	-	-
**CI_E-37A**	B-6, 1.50, saco 25	0–0.5 y	Ribs	-	-
**CI_E-39A** *	C-5, 1.18–1.48, saco 110	3 y ± 12 m	dm1/(Cr_c_)	14.5-17w - 6m	14.5-17w - 6m
			M1/(Cr_c_)	30w - 3y	30w - 3y
			Ribs	-	-
**CI_E-40A** *	C-5, 6, D-5, 6, 1.04–1.73, saco 43	9 m ± 3m	dm2 (Cr_3/4-c_)	16–23.5w - 11m	16–23.5w - 9m
			Ribs	-	-
**CI_E-41A**	D-4, 1.33–1.64, saco 102	1.5–2.5 y	Ribs	-	-
**CI_E-49A** *	A-3, 4, B-3, 3.0–3.79, saco 26	6 m ± 3m / 9 m ± 3m	di/(Cr_c_)	13-16w - 2.5m	13-16w - 2.5m
			dm1/(Cr_c_)	14.5-17w - 6m	14.5-17w - 6m
			dm2/(C_1/2-1/4_)	16–23.5w - 11m	16–23.5w - 9m
			Ribs	-	-
**CI_E-50A**	D-2, 3, 2.6–3.08, saco 167	0–0.5 y	Ribs	-	-
**CI_E-51A**	C-4, 1.8, saco 72	1y ± 4m	dm2/(Cr_c_)	16–23.5w - 11m	16–23.5w - 11m
**CI_I22**	Isolated remains	≥ 5 y	M1/(Cr_c_)	30w - 3y	30w - 3y
**CI_I23**	Isolated remains	≥ 7 y	M1/(Cr_c_)	30w - 3y	30w - 3y
**CI_I25**	Isolated remains	> 10 y	M1/(Cr_c_)	30w - 3y	30w - 3y
**CI_I26**	Isolated remains	≥ 9 y	M1/(Cr_c_)	30w - 3y	30w - 3y
**CI_I27**	Isolated remains	4 y ± 12 m	M1/(Cr_c_)	30w - 3y	30w - 3y
**CI_I28**	Isolated remains	4 y ± 12 m	M1/(Cr_c_)	30w - 3y	30w - 3y
**CI_I29**	Isolated remains	> 10 y	M1/(Cr_c_)	30w - 3y	30w - 3y
**CI_I31**	Isolated remains	≥ 7 y	M1/(Cr_c_)	30w - 3y	30w - 3y
**CI_I40**	Isolated remains	> 10 y	M1/(Cr_c_)	30w - 3y	30w - 3y
**CI_I41**	Isolated remains	5 y ± 16 m	M1/(Cr_c_)	30w - 3y	30w - 3y
**CI_I43**	Isolated remains	> 10 y	M1/(Cr_c_)	30w - 3y	30w - 3y

^1^ Crown formation stages were determined following Moorrees et al. [73] and are included within a parenthesis. di: Deciduous incisor, dc: deciduous canine, dm1: deciduous first molar, dm2: deciduous second molar, M1: First permanent molar, C: Permanent canine.

^2^Crown formation period refers to the period in which the tooth crown is formed in humans.

^3^ It refers to the specific time in which the crown was formed in the individual under study (based on crown development). Please note that if the individual died before the time at which crown completes, crown formation period and age at crown formation will differ. w: weeks, m: months, y: years.

*Individuals included in the multi-isotope model.

The sampled teeth primarily covered the period of crown formation between 13 weeks after fertilization and 3 years of age [69] (Table 1). Infant deciduous incisors (di) are expected to primarily represent the δ^18^O_en_ values of their mothers since at least 60–80% of their crown is formed as a fetus [69]. In the absence of adult tissues, the δ^18^O values of deciduous incisors were used as a proxy of the local isotope ranges. Enamel from deciduous canines (dc) starts forming between 15–18 weeks after fertilization and completes at nine months postnatal. The first deciduous molar (dm1) crown starts mineralizing 14.5–17 weeks after fertilization and completes at around six months after birth. The enamel of second deciduous molars (dc2) starts forming at 16–23.5 weeks after fertilization and completes at around 11 months postnatal. Permanent canine tooth enamel begins mineralizing between 4–5 months after birth, a process that completes at around six years of age. Finally, first permanent molars start mineralizing at around 30 weeks after fertilization, with crown formation complete at around three years [69]. Enamel powder was collected as close to the cervical portion of the tooth as possible, in order to target the later portion of the crown formation process [70]. Non-adult ages at death, and the age represented by the different tissues, were estimated by combining dental eruption [57], long bone lengths [71, 72] and the stages at crown formation [73, 74]. The age category 0–0.5 grouped individuals whose age is likely toward 3–6 months (dental development and/or long bones lengths) while newborn refer to individuals that are closer to the time at birth. Some isolated teeth’s roots were fragmented, possibly leading to inaccuracies in age at death assessments (no relevant for the results of this study). Different stable isotope systems were combined only for those tissues (tooth vs. ribs) formed at similar ages (when crown formation completes close to the age at death—see Table 1).

### Stable isotopes analysis

Sample processing was conducted at the Laboratory for Archaeological Chemistry, Faculty of Archaeology, Leiden University. Prior to sample extraction for isotope analyses, teeth were cleaned by sonicating them in distilled water for 60 minutes. After drying, enamel was extracted with a pre-cleaned, diamond-tipped drill bit attached to a hand-held drill. Circa 5 mg of powdered enamel was removed from the cervical region of the dental crown and then placed in pre-cleaned sampling tubes. Enamel extracts were pre-treated using a modified version of the protocol of [75]. This involved an initial bleaching step in a 2.5% sodium hypochlorite (NaOCl) solution, followed by thorough rinsing. The enamel samples were then treated with a 1M calcium acetate buffered acetic acid solution, rinsed to neutral and dried down. Bone collagen was extracted using a modified version of the Longin method [76]. Individual rib elements were ground in a mortar and pestle and placed into pre-cleaned sampling tubes. Samples were then demineralized in 0.6 M HCl for several days at 4° C with regular agitation, rinsed to neutral, then treated with 0.125 M NaOH for 20 hours at 20° C, and rinsed to neutral again. Collagen samples were gelatinized in 0.001 M HCl at 80° C for 3 days, purified with ezee filters (Elkay), frozen and freeze dried.

Isotopic analyses of collagen and enamel samples were conducted at the Stable Isotope Lab, Faculty of Science, Vrije Universiteit Amsterdam. Collagen carbon and nitrogen isotopes were measured on a Thermo Quest IRMS Delta XP plus interfaced with a Flash elemental analyzer. International standards (USGS40 and USGS 41, and IAEA-310(A) and IAEA-NO3) were used to monitor stability and for sample calibration. Enamel carbon and oxygen isotopes were measured on a Finnigan DeltaPlus IRMS connected with a Gasbench II universal automated interface. The isotope results are reported in the δ notation in parts per thousand (‰) relative to the international PDB (carbon and oxygen) and AIR (nitrogen) standards. Typical analytical uncertainty for both collagen δ^13^C and δ^15^N average < 0.2‰, and for enamel δ^13^C and δ^18^O < 0.15‰.

### Bayesian models and statistical analysis

Nitrogen stable isotope results from rib collagen (δ^15^N) were processed by using the WARN model (Weaning Age Reconstruction with Nitrogen isotope analysis [24]). This model accounts for changes in the turnover rates of bone collagen and provides age estimates of weaning within the framework of approximate Bayesian computation (ABC). Models to reconstruct BWPs in archaeology have assumed that the mean δ^15^N collagen values (±1SD) of the female individuals of a specific site is a good proxy for maternal δ^15^N. Thus, as the placenta is the source of all nutrition to fetuses during pregnancy, the mother and fetus are assumed to have similar stable isotope values at birth [77]. However, higher infant δ^15^N values have been reported in paired infant-mother hair samples [31, 78]. This offset may be related to a dietary or physiological changes in mother δ^15^N values during pregnancy, suggesting caution when using the δ^15^N adult female values as a baseline. In this case, adult female individuals were not available for sampling. Therefore, maternal δ^15^N values were assumed to be similar to the ones found in the newborns since it takes time for the signal of the postnatal food intake to be reflected in infant bones [24]. In the WARN model, the enrichment factor and the δ^15^N values of weaning foods are target parameters to be estimated, accounting for potential sources of intra-population variability. However, we acknowledge that using δ^15^N values of newborns as a baseline for maternal values has limitations that will be taken into account to analyze the implications of the results presented here.

Outputs of the WARN model simulation includes maximum density estimators and posterior probabilities for: the start of weaning (t_1_, the age at which food other than mother’s milk is first added to the infant’s diet), the end of weaning (t_2_, when breast milk is no longer provided), as well as the δ^15^N value (δ^15^N_wnfood_) of weaning foods, and E, the nitrogen isotope enrichment between mother and child [24]. Bayesian modeling approaches [79] are being increasingly used for dietary reconstructions [47], including BWPs [8, 9, 22, 24]. They offer advantages over *ad hoc* explanations since conclusions are based on probabilities rather than the use of fixed offsets to account for the difference in δ^15^N between bone collagen and diet.

WARN model results were used to partition individuals into three age cohorts for the purposes of subsequent statistical comparisons of δ^15^N and δ^13^C_co_. The first cohort included individuals whose age at death was less than the t_1_ value, the second comprised juveniles who died during the weaning process (age at death between t_1_ and t_2_), and the third cohort included those juveniles with ages greater than the value of t_2_. For comparison of the δ^18^O_en_ and δ^13^C_en_ values, samples were divided in four cohorts according to the age range at which dental tissues were formed: birth (tissues formed mainly in utero), first six months, six months to one year and more than two years.

We used a one-way ANOVA (F) with a Tukey-Kramer post-hoc test to assess differences among age ranges for variables that were normally distributed (δ^15^N: W = 0.93, p = 0.12; δ^13^C_co_: W = 0.98, p = 0.88; δ^13^C_en_: W = 0.99, p = 0.94). Since oxygen was not normally distributed (δ^18^O_en_: W = 0.95, p = 0.03), comparisons among ages ranges were performed by using a Kruskal Wallis test. Statistical significance was set at α = 0.05 for all tests performed.

All data underlying the findings described are fully available within this manuscript and can be found in the results section and Tables.

## Results

### Collagen: δ^15^N, δ^13^C_co_ and WARN model parameters

Nitrogen (δ^15^N) and carbon (δ^13^C_co_) stable isotope values of infants and children from the archaeological site of Bacuranao I are summarized in Table 2. All individuals, except CI_E-12, had a C/N ratio within the acceptable parameters and consequently, they were considered in this study.

**Table 2:** Nitrogen (δ^15^N) and carbon (δ^13^C_co_) stable isotope values of Bacuranao I infants and children.

ID	Age at tissue formation	Tissues	δ^15^N	δ^13^C_co_	%Col yield	%C	%N	C/N
From 0 to 6 months								
CI_E-25A*	Newborn	Ribs	10.2	-19.3	73	17	6	3.08
CI_E-37A*	Newborn	Ribs	9.6	-20.6	77	20	7	3.35
CI_E-8A	0–0.5 m	Ribs	10.5	-19.7	22	24	8	3.51
CI_E-18A	0–0.5 m	Ribs	10.1	-19.5	40	20	7	3.49
CI_E-19A	0–0.5 m	Ribs	11.8	-19.8	53	24	8	3.38
CI_E-22A	0–0.5 m	Ribs	11.2	-19.1	33	41	15	3.20
CI_E-23A	0–0.5 m	Ribs	12.4	-18.6	26	35	14	2.90
CI_E-28A	0–0.5 m	Ribs	12.1	-18.2	34	27	11	2.96
CI_E-29A	0–0.5 m	Ribs	12.5	-17.7	27	38	15	2.95
CI_E-30A	0–0.5 m	Ribs	12.6	-17.9	31	37	15	2.92
CI_E-31A	0–0.5 m	Ribs	10.6	-18.1	34	31	11	3.16
CI_E-33A	0–0.5 m	Ribs	12.8	-19.5	31	34	13	3.11
CI_E-34A	0–0.5 m	Ribs	10.3	-19.0	34	36	13	3.22
CI_E-50A	0–0.5 m	Ribs	12.2	-18.3	25	36	14	3.01
Mean			**11.3**	**-18.9**				**3.16**
SD			**1.1**	**0.8**				**0.21**
Max			**12.8**	**-17.7**				**3.51**
Min			**9.6**	**-20.6**				**2.9**
From 6 months to 1.5 years								
CI_E-49A	0.5–1.0 y	Ribs	12.6	-18.8	26	39	16	2.89
CI_E-35A	0.5–1.5 y	Ribs	11.8	-18.2	51	31	11	3.17
CI_E-36A	0.5–1.5 y	Ribs	13.4	-17.9	28	40	16	3.01
CI_E-40A	0.5–1.5 y	Ribs	11.7	-17.6	16	40	15	3.01
Mean			**12.4**	**-18.1**				**3.02**
SD			**0.8**	**0.5**				**0.11**
Max			**13.4**	**-17.5**				**3.17**
Min			**11.7**	**-18.8**				**2.89**
From 1.5 to 3.5 years								
CI_E-21A	1.5–2.5 y	Ribs	10.62	-19.1	14	40	15	3.17
CI_E-27A	1.5–2.5 y	Ribs	7.97	-19.5	24	21	8	3.11
CI_E-41A	1.5–2.5 y	Ribs	11.2	-18.8	22	41	16	2.95
CI_E-39A	2.5–3.5 y	Ribs	10.1	-18.4	24	76	29	3.03
Mean			**10.0**	**-19.0**				**3.07**
SD			**1.4**	**0.5**				**0.1**
Max			**11.2**	**-18.4**				**3.17**
Min			**8.0**	**-19.5**				**2.95**

*The Nitrogen values of these individuals were used as a proxy of female values (Ave: 9.9± 0.4) for the WARN model.

**Fig 3:**
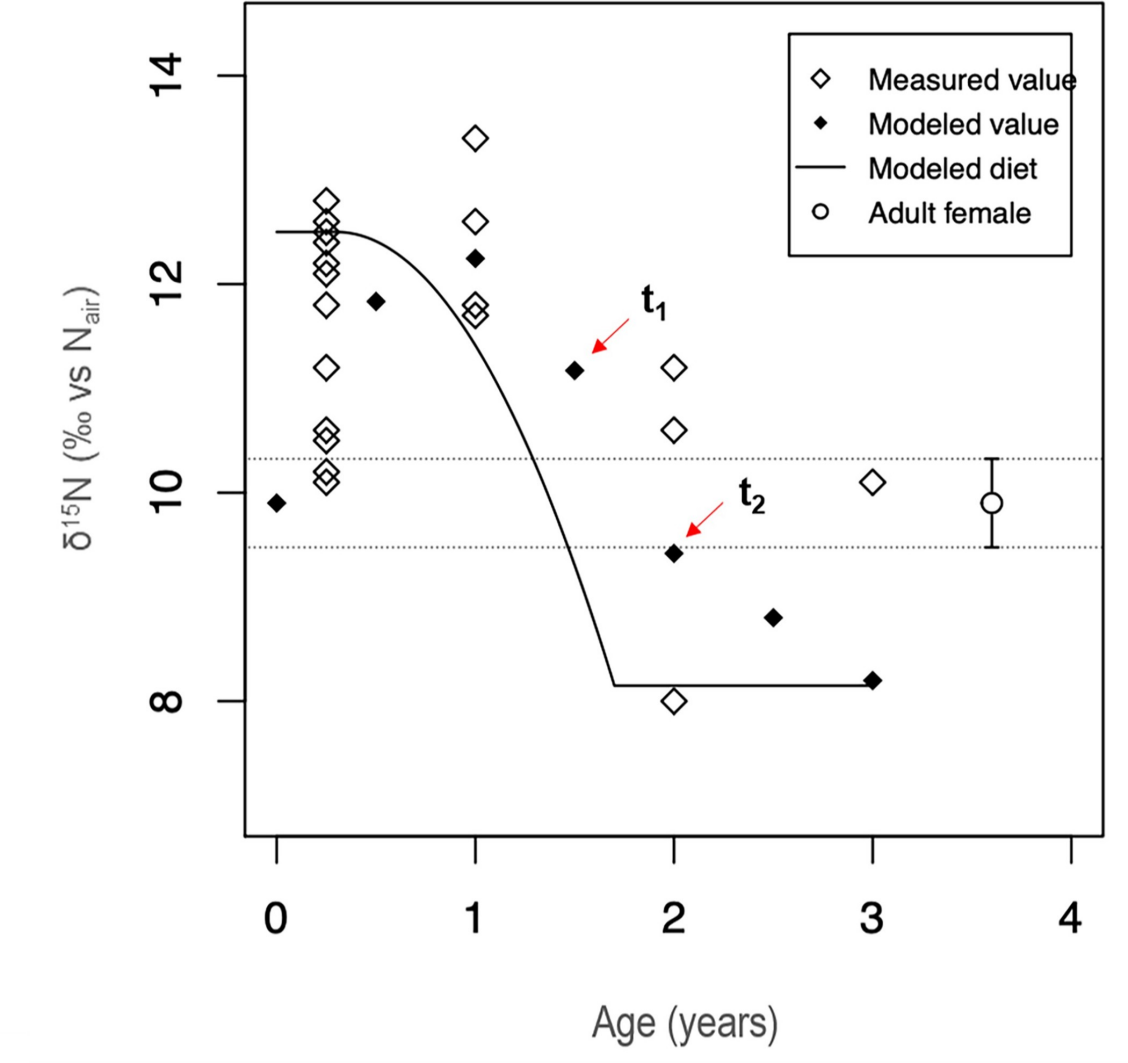
Transverse comparison of δ^15^N (‰ Air) values by infants/children age calculated from reconstructed maximum density estimators (MDEs) (Tsutaya and Yoneda, 2013). The estimated mean and SD range for adult females and all non-adults are indicated with open circles and diamonds, respectively. t_1_: start of weaning, t_2_: end of weaning.

Nitrogen values were found to be highly variable during the first six months of life (Fig 3), ranging from 9.6 to 12.8‰ (Ave: 11.3 ± 1.1‰) (Table 2). Infants between six months of age and 1.5 years still showed high δ^15^N values (Ave: 12.4 ± 0.8‰; min: 11.7‰; max: 13.4‰) in comparison to the female estimated values (9.9 ± 0.4‰). After 1.5 years, δ^15^N values are also variable, ranging from 8.0 to 11.2‰ (Ave: 10.0 ± 1.4‰) (Table 2).

**Fig 4:**
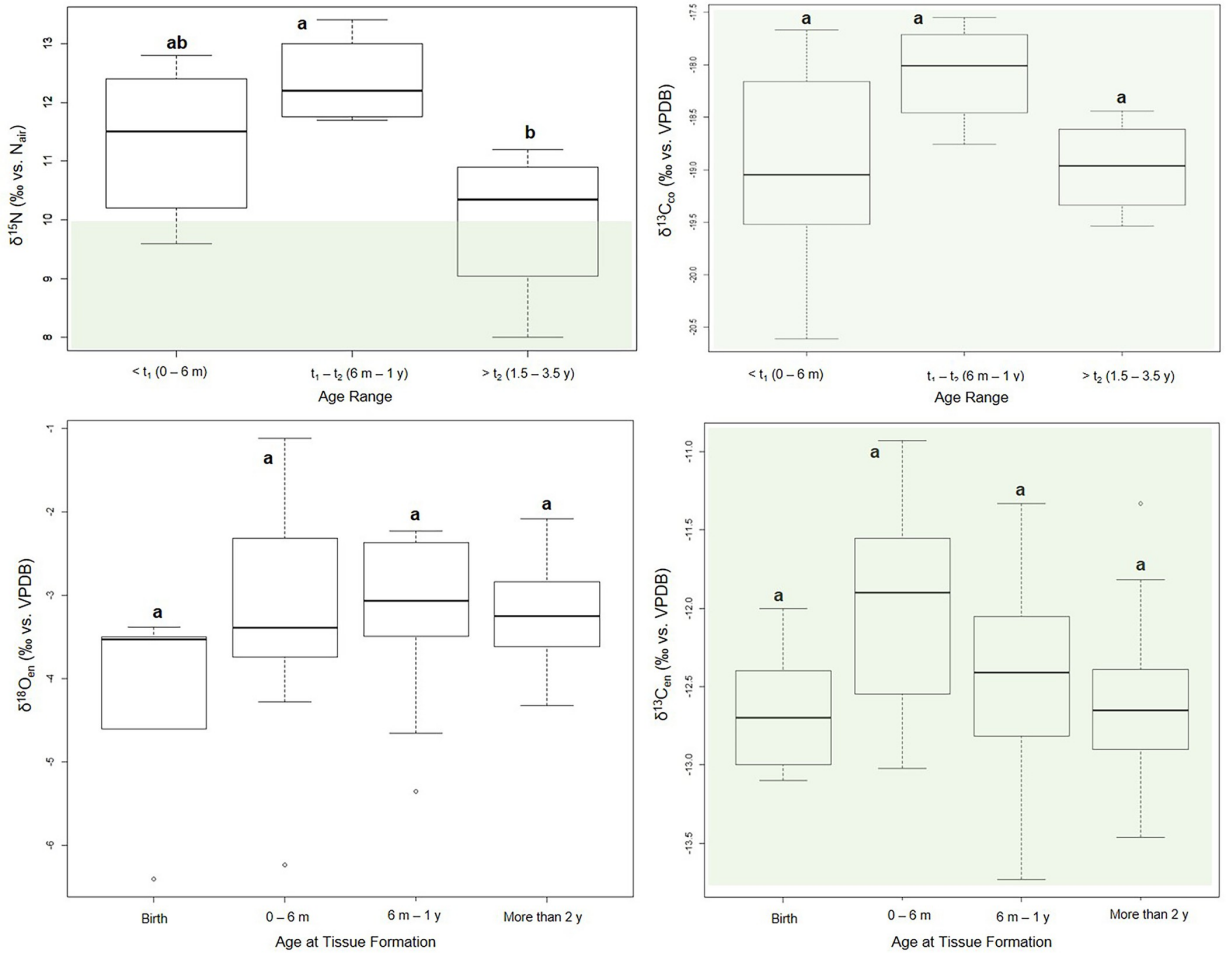
δ^15^N, δ^13^C_co_, δ^18^O, δ^13^C_en_ isotopic variations among age ranges of infants/children from Bacuranao I. Different letters (‘a’ and ‘b’) mean statistically significant differences.

A visual inspection of modeled values suggests that δ^15^N started to deplete after one year, and reached the estimated female range at roughly 3 years of age (Fig 3). The WARN model maximum density estimators (MDE) suggested that the age at the start of weaning (t_1_) occurred soon after 3 months (0.3 years) with a 0.95% credibility internal between 0 and 0.7 years. The age at cessation of weaning (t_2_) was found to be between 1.3 and 2.0 years (MDE: 1.7 years). Significant variations were found among age ranges (F = 4.64, df = 2, p = 0.02) due to the statistically significant difference (p = 0.02) between infants from 0.5–1.5 years (t_1_-t_2_: infants in the weaning process) and the ones from 1.5 to 3.5 years of age (weaned children according to WARN) (Fig 4). The enrichment of infant’s δ^15^N values was 2.6‰ (MDE; 95% CI: 1.9–3.3‰). Collagen nitrogen entirely derived from weaning foods had an MDE of 8.2‰ (95% CI: 7.5–8.8‰).

Carbon collagen values during the first six months of life ranged from -17.7 to -20.6‰ (Ave: -18.9 ± 0.8‰). Infant’s δ^13^C_co_ values were elevated by approximately 1‰ from six months to 1.5 years (Ave: -18.1 ± 0.5‰), ranging between -17.5‰ and -18.8‰. From 1.5 to 3.5 years (Ave: -19.0 ± 0.5‰; max: -18.4‰; min: -19.5‰) δ^13^C_co_ values decreased. No statistically significant variations in δ^13^C_co_ were found among age ranges (F = 2.21, df = 2, p = 0.14) (Fig 4).

### Enamel: δ^18^O and δ^13^C_en_ values

Oxygen (δ^18^O) and carbon enamel (δ^13^C_en_) stable isotope values are summarized in Table 3. The δ^18^O of deciduous incisors (*di*) ranged from -6.4‰ to -3.4‰ (Ave: -4.3 ± 1.3‰). Tissues formed during the first six months of life showed values between -6.2‰ and -1.1‰ (Ave: -3.2 ± 1.4‰), while enamel formed between six months and one year showed less variation (Ave: -3.2 ± 1.0‰; Max: -2.2‰; Min: -5.4‰). Enamel formed after two years of age ranged from -4.3‰ to -2.1‰ (Ave: -3.3 ± 0.6‰) (Table 3). Higher enrichment was observed during the first six months of life, but no statistically significant differences were found between age ranges (H = 4.61, df = 3, p = 0.20) (Fig 4).

**Table 3:** Oxygen and Carbon enamel values of individuals from Bacuranao I (Mayabeque, Cuba).

ID	Teeth	Age at Tissue Formation	δ^18^O Average	δ^18^O SD	δ^13^C_en_ Average	δ^13^C_en_ SD
Birth (n = 5)						
CI_E-6A	di	13-16w - 2.5m	-4.6	0.08	-12.7	0.06
CI_E-21A	di	13-16w - 2.5m	-6.4	0.05	-12.4	0.04
CI_E-30A	di	13-16w - 2.5m	-3.5	0.10	-12.0	0.10
CI_E-35A	di	13-16w - 2.5m	-3.4	0.08	-13.0	0.09
CI_E-49A	di	13-16w - 2.5m	-3.5	0.12	-13.1	0.08
Mean			**-4.28**		**-12.63**	
SD			**1.28**		**0.45**	
Min			**-6.40**		**-13.10**	
Max			**-3.38**		**-12.00**	
0–6 Months (n = 11)						
CI_E-34A	dm1	14.5-17w - 3m	-1.68	0.10	-12.32	0.05
CI_E-1	dm1	14.5-17w - 6m	-1.12	0.10	-12.84	0.09
CI_E-31A	dm1	14.5-17w - 6m	-3.39	0.15	-11.90	0.09
CI_E-36A	dm1	14.5-17w - 6m	-6.23	0.14	-12.77	0.11
CI_E-39A	dm1	14.5-17w - 6m	-4.28	0.08	-11.78	0.06
CI_E-49A	dm1	14.5-17w - 6m	-2.42	0.12	-13.02	0.07
CI_E-29A	dc	15-18w - 6m	-1.46	0.07	-11.87	0.05
CI_E-29A	dm2	16–23.5w - 6m	-2.96	0.11	-10.93	0.06
CI_E-29A	dm2	16–23.5w - 6m	-5.35	0.08	-12.00	0.07
CI_E-6A	dm2	16–23.5w - 6m	-4.98	0.09	-12.73	0.08
CI_E-6A	M1	30w – 6m	-2.93	0.09	-11.57	0.05
CI_E-21B	dm2	16–23.5w – 6m	-3.53	0.08	-11.51	0.08
CI_E-28A	dm2	16–23.5w - 6m	-3.39	0.06	-11.36	0.05
CI_E-30A	dm2	16–23.5w - 6m	-2.43	0.07	-10.93	0.06
Mean			**-3.33**		**-12.00**	
SD			**1.49**		**0.65**	
Min			**-6.23**		**-13.02**	
Max			**-1.12**		**-10.93**	
6 Months– 1 Year (n = 11)						
CI_E-1C	dc	15-18w – 9m	-2.34	0.05	-12.41	0.06
CI_E-21C	dc	15-18w – 9m	-2.40	0.15	-12.31	0.17
CI_E-49A	dm2	16–23.5w - 9m	-2.92	0.06	-12.88	0.05
CI_E-40A	dm2	16–23.5w – 11m	-2.36	0.07	-11.33	0.03
CI_E-1	dm2	16–23.5w – 11m	-2.23	0.11	-11.72	0.10
CI_E-1D	dm2	16–23.5w - 11m	-3.49	0.06	-13.40	0.04
CI_E-21D	dm2	16–23.5w – 11m	-2.38	0.36	-11.79	0.30
CI_E-30	dm2	16–23.5w - 11m	-4.65	0.13	-13.73	0.16
CI_E-35A	dm2	16–23.5w - 11m	-4.68	0.16	-12.63	0.12
CI_E-35A	dm2	16–23.5w - 11m	-2.32	0.12	-12.18	0.09
CI_E-36A	dm2	16–23.5w - 11m	-3.28	0.10	-12.67	0.13
CI_E-51A	dm2	16–23.5w - 11m	-3.21	0.05	-12.75	0.06
Mean			**-2.98**		**-12.49**	
SD			**0.74**		**0.71**	
Min			**-4.68**		**-13.73**	
Max			**-2.23**		**-11.33**	
More than 2 Years (n = 17)						
CI_E-21A	M1	30w – 2y	-3.18	0.09	-11.33	0.06
CI_E-1C	M1	30w - 3y	-3.25	0.13	-12.96	0.05
CI_E-21E	M1	30w – 3y	-2.84	0.09	-12.84	0.08
CI_E-21F	M1	30w – 3y	-2.97	0.18	-13.03	0.13
CI_E-39A	M1	30w - 3y	-3.98	0.14	-12.48	0.18
CI_122	Full	30w - 3y	-2.75	0.09	-11.82	0.09
CI_123	Full	30w - 3y	-3.65	0.12	-12.71	0.11
CI_125	Full	30w - 3y	-3.62	0.13	-12.57	0.09
CI_126	Full	30w - 3y	-3.34	0.30	-12.90	0.22
CI_127	Full	30w - 3y	-4.32	0.14	-12.53	0.09
CI_128	Full	30w - 3y	-3.55	0.20	-12.30	0.21
CI_129	Full	30w - 3y	-3.36	0.20	-12.65	0.15
CI_131	Full	30w - 3y	-2.21	0.09	-12.39	0.10
CI_140	Full	30w - 3y	-4.28	0.22	-13.46	0.20
CI_141	Full	30w - 3y	-3.24	0.13	-12.24	0.04
CI_143	Full	30w - 3y	-2.08	0.07	-13.09	0.10
AdultoB-E-1	C	4-5m - 6y	-2.81	0.16	-12.82	0.09
Mean			**-3.26**		**-12.60**	
SD			**0.63**		**0.50**	
Min			**-4.32**		**-13.46**	
Max			**-2.08**		**-11.33**	

**Fig 5:**
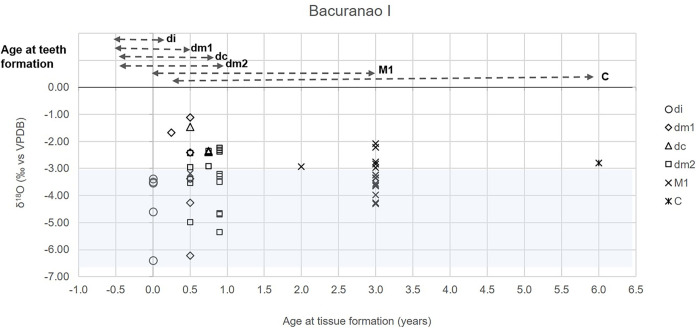
Transverse comparison of enamel oxygen stable isotope values (δ^18^O) by age at tissue formation of the Bacuranao I individuals. The blue line represents mother’s estimated range.

As explained before, the values of *di* were used as a baseline to estimate the mothers’ δ^18^O values (Ave: -4.3 ± 1.3‰). Tissues formed during the first six months of life were highly variable with some teeth being similar in their δ^18^O values to the reference baseline, while others were enriched (Fig 5). Teeth whose enamel formation was complete at around nine months were all enriched in comparison to the estimated mother’s values. After one year, most teeth had values similar to the baseline but the tissues of some individuals were still enriched (Fig 5).

The δ^13^C_en_ of *di* ranged from -13.1‰ to -12‰ (Ave: -12.6 ± 0.5‰). Tissues formed during the first six months of the individual’s life were more variable, ranging from -13.0‰ to -10.9‰ (Ave: -12.0 ± 0.7‰). Enamel formed between six months and one year showed values between -13.7‰ and -11.3‰ (Ave: -12.5 ± 0.7‰), while enamel formed after two years of age ranged from -13.5‰ to -11.3‰ with an average value similar to the female estimates (Ave: -12.6 ± 0.5‰) (Table 3). There is a tendency toward having higher average values during the first six months of life (Fig 4), although no statistically significant differences were found between age ranges (F = 2.55, df = 3, p = 0.07) (Fig 4).

### Individual variation: δ^18^O and δ^13^C_en_ values

**Fig 6:**
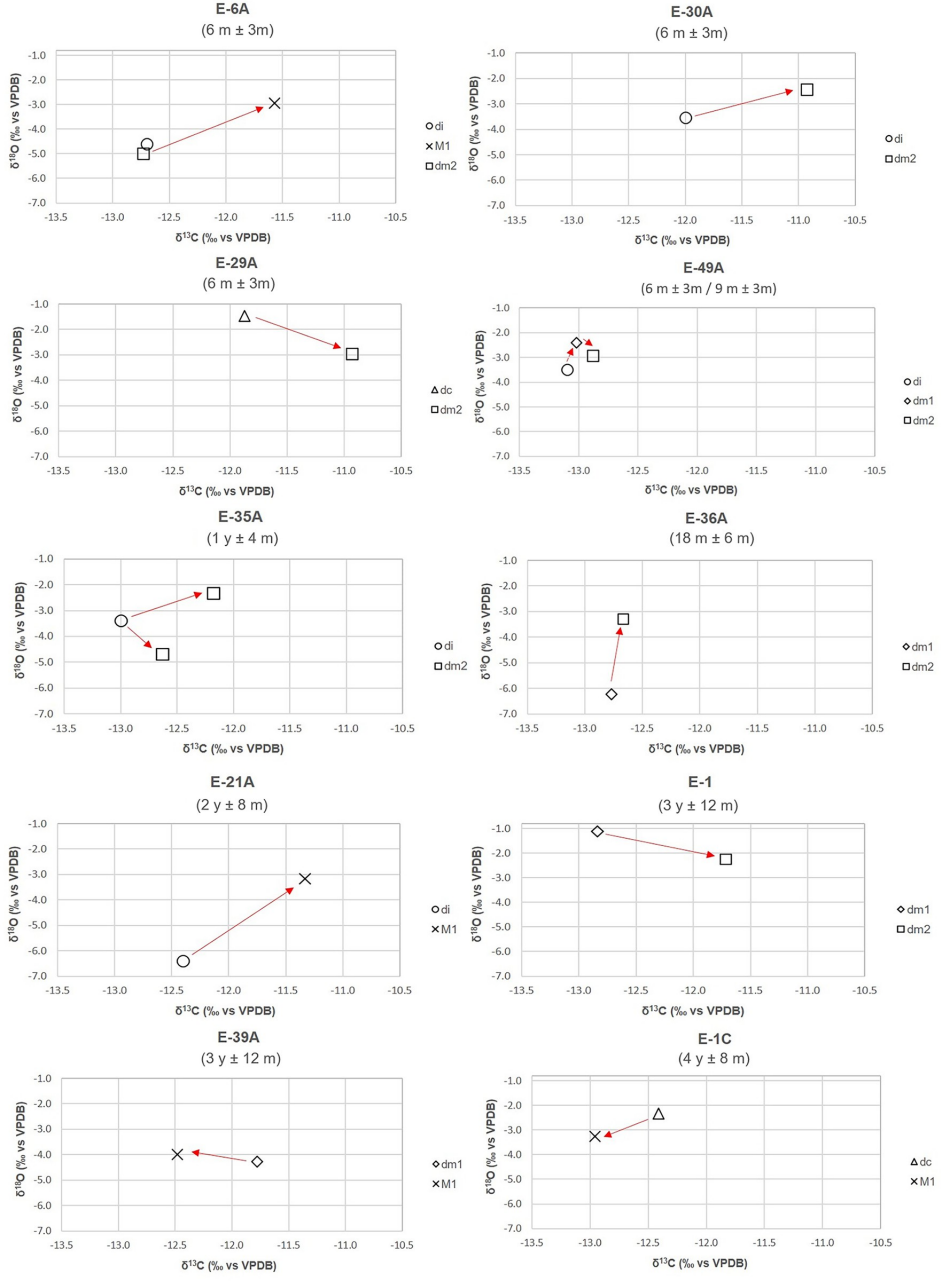
Variations in δ^18^O and δ^13^C_en_ among dental tissues of infants/children at Bacuranao I, Mayabeque, Cuba. Arrows run from earlier to later developing tooth.

Concerning intra-individual variation (between samples from the same individual), oxygen isotope values in teeth that form at different periods showed low to moderate variation in comparison to the differences observed between teeth that form at the same time period [e.g, E-35A (age at death: 1y ± 4m; Δdm2-dm2 = -2.4‰) Table 4, Fig 6]. Most deciduous and permanent molars were enriched in δ^18^O in comparison to the deciduous incisors of the same individual (Fig 6; E-6A: Δdi-M1 = -1.7‰; E-30A: Δdi-dm2 = -1.1‰; E-49A: Δdi-dm1 = -1.1‰, Δdi-dm2 = -0.6‰; E-35A: Δdi-dm2 = -1.1‰; E-21A: Δdi-M1 = -3.2‰), with the exception of the dm2 of E-6A and E-35A that were depleted +0.4 and +1.3‰, respectively (Fig 6). The δ^18^O isotopic composition of most dm2 was lower than the values of dm1 and dc of the same individuals (Fig 6; E-29A: Δdc-dm2 = +1.5‰; E-49A: Δdm1-dm2 = +0.5‰; E-1: Δdm1-dm2 = +1.1‰), with the exception of E-36A where dm2 was enriched 3.0‰ in comparison to the dm1. The isotopic composition of the first permanent molars (M1s) was enriched in oxygen in comparison to di, dm1 and dm2 (Fig 6, E-6A: Δdi-M1 = -1.7‰; Δdm2-M1 = -2.0‰; E-21A: Δdi-M1 = -3.2‰; E-39A: Δdm1-M1 = -0.3‰) and depleted in comparison to dc (Fig 6, E-1C: Δdc-M1 = +0.9‰).

**Table 4:** Variations in oxygen and carbon stable isotope values of different teeth from the same individuals at Bacuranao I, Mayabeque, Cuba.

ID	Teeth	Age at Tissue Formation	δ^18^O	δ^18^O	δ^13^C_en_	δ^13^C_en_
			Ave (‰)	SD	Ave (‰)	SD
CI_E-1	dm1	14.5-17w – 6m	-1.1		12.8	
	dm2	16–23.5w – 11m	-2.2		-11.7	
**Δ dm1-dm2**			**+1.1**		**-1.1**	
CI_E-1C	dc	15-18w - 9m	-2.3		-12.4	
	M1	30w - 3y	-3.3		-13.0	
**Δ dc-M1**			**+0.9**		**+0.6**	
CI_E-6A	di	13-16w - 2.5m	-4.6	0.08	-12.7	0.06
	dm2	16–23.5w - 6m	-4.98	0.09	-12.73	0.08
	M1	30w – 6m	-2.93	0.09	-11.57	0.05
**Δ di-dm2**			**+0.4**		**0.0**	
**Δ di-M1**			**-1.7**		**-1.1**	
**Δ dm2-M1** *			**-2.0**		**-1.2**	
CI_E-21A	di	13-16w - 2.5m	-6.4	0.05	-12.4	0.04
	M1	30w – 2y	-3.18	0.09	-11.33	0.06
**Δ di-M1**			**-3.2**		**-1.1**	
CI_E-29A	dc	15-18w - 6m	-1.46	0.07	-11.87	0.05
	dm2	16–23.5w - 6m	-2.96	0.11	-10.93	0.06
**Δ dc-dm2** *			**+1.5**		**-0.9**	
CI_E-30A	di	13-16w - 2.5m	-3.5	0.10	-12.0	0.10
	dm2	16–23.5w - 6m	-2.43	0.07	-10.93	0.06
**Δ di-dm2**			**-1.1**		**-1.1**	
CI_E-35A	di	13-16w - 2.5m	-3.4	0.08	-13.0	0.09
	dm2	16–23.5w - 11m	-4.68	0.16	-12.63	0.12
	dm2	16–23.5w - 11m	-2.32	0.12	-12.18	0.09
**Δ di-dm2**			**+1.3**		**-0.4**	
**Δ di-dm2**			**-1.1**		**-0.8**	
**Δ dm2-dm2** *			**-2.4**		**-0.5**	
CI_E-36A	dm1	14.5-17w - 6m	-6.23	0.14	-12.77	0.11
	dm2	16–23.5w - 11m	-3.28	0.10	-12.67	0.13
**Δ dm1-dm2**			**-3.0**		**+0.7**	
CI_E-39A	dm1	14.5-17w - 6m	-4.28	0.08	-11.78	0.06
	M1	30w - 3y	-3.98	0.14	-12.48	0.18
**Δ dm1-M1**			**-0.3**		**0.7**	
CI_E-49A	di	13-16w – 2.5m	-3.5	0.12	-13.1	0.08
	dm1	14.5-17w - 6m	-2.42	0.12	-13.02	0.07
	dm2	16–23.5w - 9m	-2.92	0.06	-12.88	0.05
**Δ di-dm1**			**-1.1**		**-0.1**	
**Δ di-dm2**			**-0.6**		**-0.2**	
**Δ dm1-dm2** *			**+0.5**		**-0.1**	

* Pairs of teeth expected to have been formed at around the same time periods.

With respect to intra-individual variation, differences in δ^13^C_en_ among deciduous teeth from the same individual were low for many pairwise combinations (Fig 6, E-6A: Δdi-dm2 = 0.0‰; E-35A: Δdi-dm2 = -0.4‰; E-36A: Δdm1-dm2 = -0.7‰; E-49A: Δdi-dm1 = -0.1‰; Δdi-dm2 = -0.2‰; Δdm1-dm2 = -0.1‰), in comparison to the difference observed between two different dm2 (expected to be formed at the same time) from one individual (E-35A: Δdm2-dm2 = 0.5‰). However, a difference of around 1.0‰ was observed among deciduous teeth in some infants/children (Fig 6, E-30A: Δdi-dm2 = -1.1‰; E-29A: Δdc-dm2 = -0.9‰; E-35A: Δdi-dm2 = -0.8‰; E-1: Δdm1-dm2 = -1.1‰). The first permanent molars were enriched around 1.1‰ for infants who died before three years (Fig 6, E-6A: Δdi-M1 = -1.1‰; Δ dm2 –M1 = -1.2‰; E-21A: Δdi-M1 = - 1.1‰), but depleted for older children (Fig 6, E-39A: Δ dm1 –M1 = +0.7‰; E-1C: Δ dc–M1 = +0.6‰).

### Multi-isotope system model: Combining δ^18^O_en_, δ^15^N, δ^13^C_en_ and δ^13^C_co_ values to detect the start and the end of weaning

**Fig 7:**
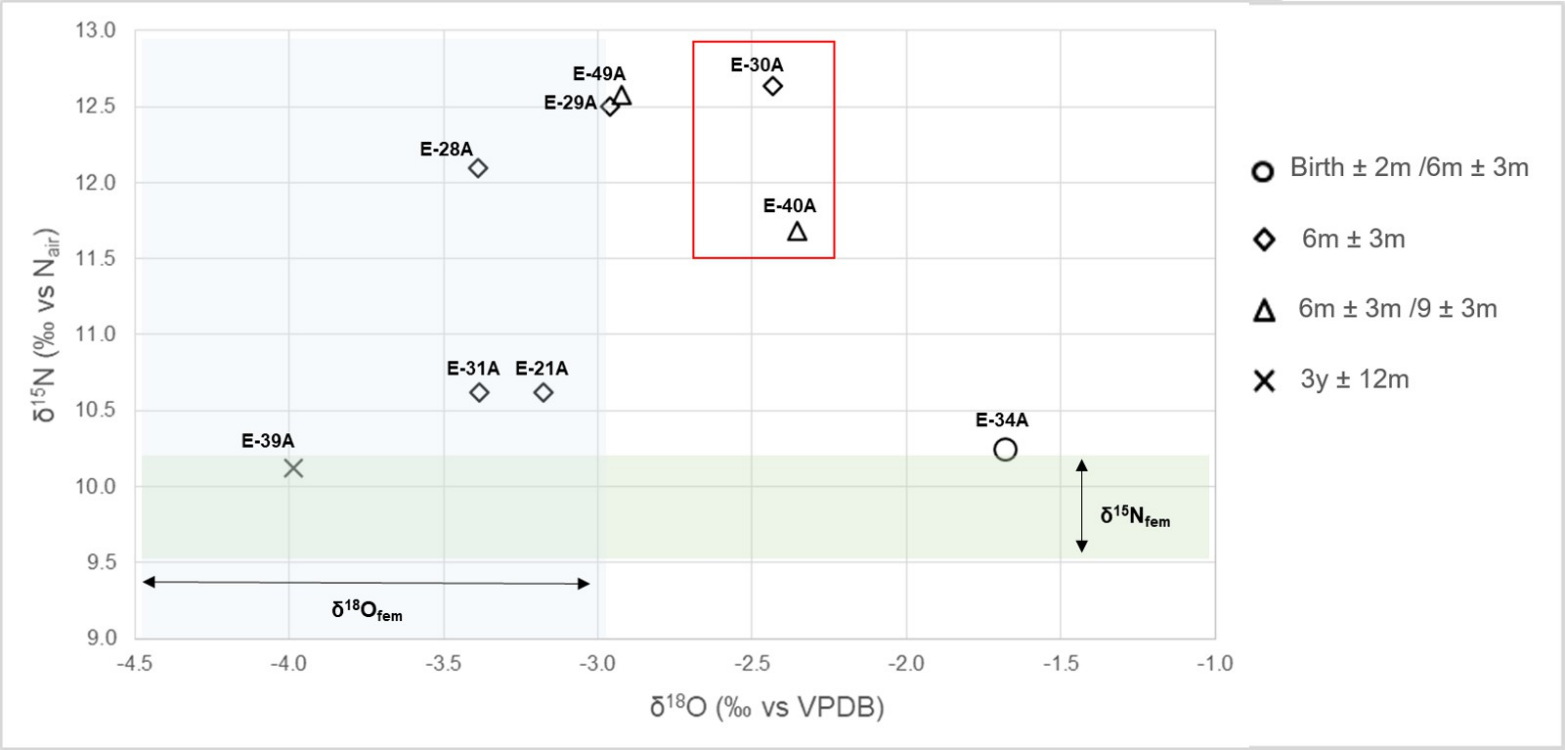
Rib collagen nitrogen (δ^15^N) and tooth enamel oxygen (δ^18^O) stable isotope composition formed at similar ages in infants and children from Bacuranao I, Mayabeque, Cuba. Shadow areas represent mother’s estimated δ^18^O and δ^15^N values.

The combination of isotope values from tissues formed at a similar time period (ribs and enamel formed close to the age at death, see Table 1), from the same individuals, showed that most individuals enriched in δ^15^N had oxygen isotope values higher than the estimated mother’s values average (from left to right: E-29A, E-49A, E-30A and E-40A) (Fig 7). Individual E-34 (~ 3 months) was enriched in δ^18^O but depleted in δ^15^N. One individual (E-28A: ~ six months) was enriched in δ^15^N but showed δ^18^O values within the mother’s estimated range. The older individual from the group (E-39A: ~ 3 y) was depleted in both δ^18^O and δ^15^N relative to the other sampled individuals with values within the range of the mother’s estimated values (Fig 7).

**Fig 8:**
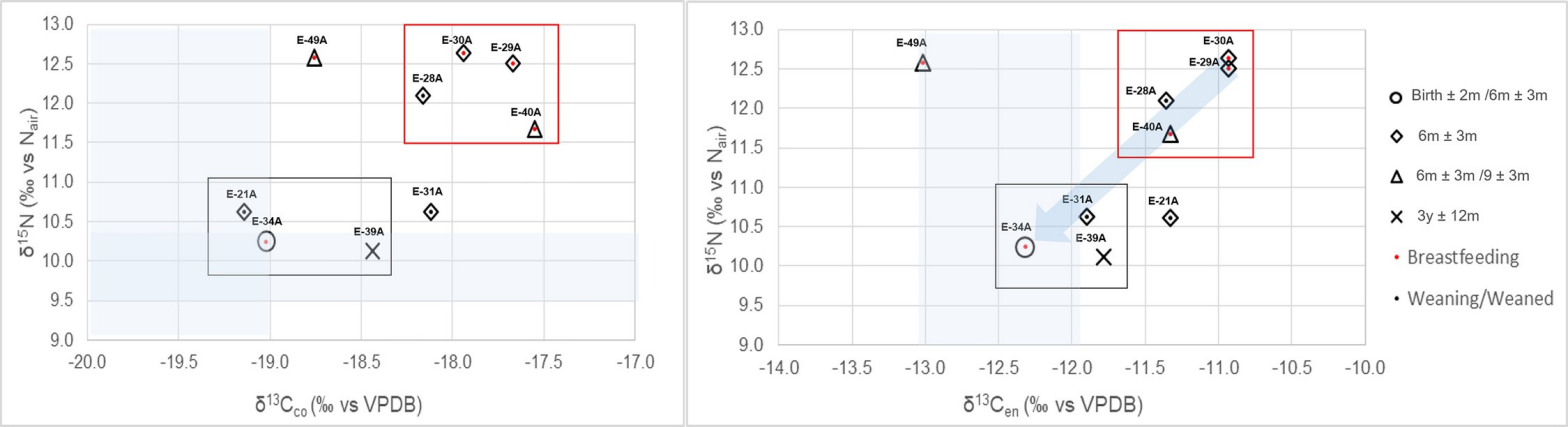
Nitrogen (δ^15^N) and carbon (δ^13^C_co_, δ^13^C_en_) stable isotope composition of tissues formed at similar ages in infants and children from Bacuranao I, Mayabeque, Cuba. Shadow areas represent mother’s estimated δ^18^O and δ^13^C values. δ^15^N and δ^13^C_co_ (collagen from ribs), δ^13^C_en_ (tooth enamel).

Four out of the five individuals enriched in collagen nitrogen (E-30A, E-40A, E-29A, E-28A) cluster together in both δ^13^C_en_ and δ^13^C_co,_ while most individuals with lower δ^15^N showed more depleted δ^13^C_en_ and δ^13^C_co_ values (Fig 8). This supports that both collagen and enamel carbon isotope values are useful for detecting age-related changes in dietary inputs. The combination of multiple isotope proxies derived from different tissues forming at similar time periods thus provides important insights into inter-individual variation in BWPs (see discussion section).

## Discussion

Combining multiple stable isotope systems in different tissues from the same individuals showed potential for both understanding the possible sources of intra-population variability, and refining the resolution at which events related to the weaning process can be detected in archaeological populations. The variability in stable isotope values of non-adult tissues formed at similar ages suggested different weaning trajectories among Bacuranao I’s infants, indicating that a more refined individual-based approach may be needed to understand variability in BWPs at a population level.

While the WARN model predicts that the age at the start of weaning occurred at around four months of age (MDE = 0.3 years), most non-adult tissues formed until one year of age are still enriched in both δ^15^N and δ^18^O, suggesting that breastmilk was the most important protein [25–27] and water source [36, 80] in their diets during that time. In contrast, some infants younger than a year showed no signal of breastfeeding when comparing their δ^15^N and δ^18^O values with the estimated female baseline, suggesting that external food supplements and water were introduced into their diet, even before six months. This apparent disagreement between WARN model parameters and the visual inspection of the data (e.g., Fig 3) can be influenced by several factors, including the input of the low δ^15^N values observed in the collagen of some infants who died before six months of age into the WARN model, an artifact of the inaccuracy of the female δ^15^N and δ^18^O estimated values used as a baseline, or a time lag of δ^15^N values due to turnover rates, a factor that the WARN model does incorporate to estimate its parameters [24].

The transversal analysis of our data showed higher variability in stable isotope values for tissues formed during the first six months of life, especially for δ^15^N. High variability in collagen δ^15^N values during the first few months of life has been observed in archaeological populations from the Antilles [8, 9] and more globally [24, 81]. Low δ^15^N values in newborns can occur when breastmilk isotopic signals have not been completely fixed in bone collagen due to the time needed between the new dietary protein intake and the synthesis of the new organic matrix [24, 82]. In addition, nitrogen stable isotope values may be influenced by several physiological factors, including a decrease in δ^15^N because of growth and development [83, 84], or a δ^15^N enrichment related to nutritional stress [85, 86]. It is possible that some of the individuals included to this study may be affected by one or more of these physiological factors [19], thus not representing the survivors weaning trajectory of their source population. Short term variations in stable isotope values of mothers can also be an important source of disparity among infant’s tissues at early ages. Several studies have shown differences in δ^15^N values of hair and bones between perinates and their mothers [78, 87], which can be influenced by physiological [88] and/or nutritional variations [85] in maternal δ^15^N values during pregnancy. Although estimating maternal baseline values from a few newborns can be problematic for the reasons discussed above, both the enrichment estimated by the WARN model, and the fact that δ^15^N of older infants/children (weaned) are similar to those of the baseline, suggests that the stable isotope values of the newborns used in this study as a reference are likely representative of the mother’s range of values in this sample population.

The start of weaning has been traditionally associated with the moment at which δ^15^N collagen values start to deplete [32], which occurred for tissues formed after 1 year for the sampled Bacuranao I individuals. It is unlikely that breastmilk had been the sole or main source of dietary protein of Bacuranao I infant diets until one year of age, since supplementation is important for both the infant’s proper nutrition [14, 15] and their immunological development after six months of age [16–19]. A delayed signal of supplementation in nitrogen isotope values may be associated with δ^15^N values primarily reflecting the protein source of diet [47], while typical complementary (weaning) foods are generally high in carbohydrates and low in protein [45], failing to recognize the start of solid food supplementation [8]. Ethnographic studies of the Island Caribs and the Warao of the Amacuro and Orinoco deltas have identified the use of herbal teas, fruits, and root cultigens such as sweet potatoes, yautia, and arrowroots to supplement non-adult diets [89–94]. The use of external sources of water to prepare supplements at early ages would account for the depletion in δ^18^O values observed in some individuals in Fig 5. However, potential sources of variability in δ^18^O among infants such as diverse origins and cohorts, seasonality (e.g., [52]), asymmetry in similar teeth development times [95], and breastmilk intake during the weaning process [39], introduce further complexities when drawing conclusions related to the age at the start of weaning. It is likely that the decrease of δ^15^N collagen stable isotope values after one year is signalizing the second stage of the weaning process (Fig 1: stage 3, c-d) where adult food, including proteins, are increasingly incorporated into infant diets. This is further supported by the transversal analysis of δ^13^C_co_ values that decrease after 1.5 years, suggesting that a change in the carbon source of diet (primarily protein) occurred along with the decrease in δ^15^N. This change in the protein source of diet occurred likely some months before it is possible to detect it in infant’s tissues due to delayed protein biosynthesis and bone turnover rates, making it important to use models that consider this factor (e.g., [24]) when making conclusions on specific weaning ages from the stable isotope values of bone collagen.

The analysis of individual variability in δ^18^O values, as reflected by teeth formed at different time periods in the same infants, confirmed that most other deciduous teeth were elevated approximately 1.0‰ (mode: 1.1‰) in comparison to deciduous incisors. Since an important portion of the deciduous incisor crown is formed in utero (~80% for the central di), and final mineralization occurs at around 1.5 months of life [69], di δ^18^O values should better represent mother’s δ^18^O values, because fetal tissues are expected to be in isotopic equilibrium with mother’s blood values. A δ^18^O enrichment of around 1.1‰ between adult and infants’ tissues have been previously associated with breastfeeding in comparative studies of teeth enamel [36, 37, 39, 42] and infant bone phosphates [39, 42]. The intra-individual analysis from this study also showed that most dm2 were depleted from 0.5 to 1.5‰ in comparison to dm1 (crown completes at ~ 6 months) and dc (crown completes at ~ 9 months), suggesting that external water sources may have been introduced into those infant diets by 11 months. However, the high δ^18^O variability observed (as high as 2.4‰) between a couple of dm2s (and other teeth) from the same individual, expected to have been formed at the same time period (Table 4), suggests caution with interpretations related to breastfeeding when comparing δ^18^O enamel enrichments by age at both individual and population levels. The effects of seasonality on the δ^18^O values of drinking water may lead to variations in the isotopic composition of infant tissues depending on their month/season of birth, periodicity of mineralization [96], teeth development patterns [57] and geographic origins [97]. This may explain the high δ^18^O variability observed in tissues formed during the first six months of the individual’s life (SD = 1.4‰). These sources of variability may also explain the δ^18^O values of individuals that did not conform to the patterns previously described.

The higher enrichment found in the first permanent molars (M1), in comparison to deciduous incisors and molars, would suggest that their δ^18^O composition is influenced by the consumption of breastmilk water [36, 37]. However, other factors can lead to such a 1.2 to 2.3‰ isotopic enrichment, including the consumption of food processed with modern cooking techniques [98], or the use of fermented or boiled liquids [99]. The fact that tissues formed after two years showed less variability in δ^18^O (SD = 0.6‰) than tissues formed at earlier periods may indicate that the source of oxygen at that time is more homogeneous (local water, food processed in similar ways). The enrichment of 1.0‰ observed in the M1 δ^13^C_en_ values of infants younger than three years may indicate that supplements used during the weaning process were enriched 1.0‰ in comparison to mother’s milk, and/or that a trophic enrichment because of breastfeeding [31] was still predominant in those tissues. The δ^13^C_en_ composition of M1s of older children suggest that an isotopic change toward a diet more negative in carbon, and similar to the inferred female values, had occurred by the last period of M1 formation (around 3 years) in those infants. These results are consistent with the transversal analysis of δ^13^C_en_ that indicated that tissues formed after two years showed the same average and SD as the estimated female values. Our results support the supposition that δ^18^O from M1s contain information related to the weaning process [39] and, when combined with δ^13^C_en_ in M1s from different stages of development, may help to better estimate the age at the end of weaning.

The analysis of multiple isotope systems in tissues formed at similar ages in the same individuals suggests that by three months of age, the δ^18^O enrichment is already quantifiable in dental enamel (Fig 7: E-34A). The high δ^18^O value observed in this infant (E-34A) would indicate that breastmilk was the main source of water intake. In contrast, the δ^15^N composition of E-34A is low and similar to the female estimated value, which suggests a time lag in the appearance of this process in the δ^15^N signal of bone collagen in comparison to δ^18^O in enamel. Tissues such as growing tooth enamel are expected to reflect the δ^18^O values of external water in around three weeks [100, 101], while tissues continually remodeling, such as bones [22, 102], may take longer to reflect the isotopic changes in diet. This individual also has low δ^13^C values in both collagen and enamel, suggesting that the trophic level enrichment in carbon, because of exclusive breastmilk consumption [31], may take longer than three months to be detectable in those tissues. Alternatively, the high δ^18^O values of this individual may be related to an unidentified physiological problem, since a different origin seems an unlikely source of oxygen variation (the ^87^Sr/^86^Sr values lies within 1 SD of the “population” mean [Chinique de Armas et al., forthcoming]).

Only two individuals (E-30A and E-40A), were clearly enriched in both δ^18^O and δ^15^N in comparison to the estimated mother’s range (Fig 7). Nevertheless, all individuals with high nitrogen values, including E-28A, had δ^18^O composition higher than the female estimated mean, supporting that potential sources of δ^18^O variability discussed above can account for the lower than expected δ^18^O enamel composition of their enamel. The enrichment in δ^18^O and δ^15^N values observed in the tissues of E-30A and E-40A, and probably in E-29A and E-49A as well, is consistent with exclusive consumption of breastmilk. Notably, most of them are enriched in both δ^13^C_en_ and δ^13^C_co_ values, which could be influenced by the expected 1‰ trophic level effect in carbon stable isotope values of exclusive breastfeeding infants [31, 103]. Although these elements represent distinct and independent stable isotope systems (δ^18^O: water intake, δ^15^N: dietary protein, δ^13^C_co:_ mainly dietary protein, δ^13^C_en:_ carbohydrates, lipids and proteins), they all are expected to be enriched during exclusive breastfeeding (stage 1 of the weaning process, Fig 1). The 1‰ enrichment observed in δ^13^C_en_ values of tissues of individuals expected to be in the weaning process (M1), suggest that some of the individuals with high δ^15^N values in Fig 7 may be in the first stage of the weaning process (Fig 1: stage 2), suggesting that nitrogen isotope values alone cannot accurately detect the age at the start of weaning.

Our data suggest that a gradual consistent transition in stable carbon enamel values occur from breastfeeding infants to weaned children, while nitrogen values are more variable (Fig 8). Since different physiological processes can affect δ^15^N values in infants and children [62, 81], a multi-isotope system approach may help to identify individuals affected by those processes. For instance, E-49A nitrogen isotope values would indicate exclusive breastfeeding (Fig 7), but both the carbon collagen and enamel values are consistent with those of weaned individuals (Fig 8). It is possible that this elevated nitrogen isotope value is the result of nutritional stress [85, 86], or the intake of atypical low-protein, ^13^C supplements. In both cases, it shows the limitations of relying solely on δ^15^N values in transversal studies of individuals that did not survive the weaning process.

The analysis of collagen and enamel of the same individuals suggest that δ^13^C values have similar tendencies during exclusive breastfeeding, and the first stage of the weaning process, likely because δ^13^C_co_ better reflects the values of macronutrients other than protein in conditions of low protein intake [104], as is the case of exclusive breastmilk consumption, or the intake of dietary supplements typically used at the beginning of the weaning process. It may suggest that the time lag observed in the depletion of δ^13^C_co_ average values (decrease after 1.5 years) in comparison to δ^13^C_en_ (decreases for infants older than six months) in transversal analysis is not mainly due to δ^13^C_en_ better detecting the carbohydrates sources of diet [103, 105], but due to slow turnover rates of bone collagen.

The multi-isotope analysis of different tissues in the same individuals showed that older infants/children exhibit higher variation in δ^13^C_co_ than in δ^13^C_en_, which suggests that sources of carbohydrates were less isotopically variable than sources of protein in Bacuranao I. The stable isotope values suggest that weaning and adult food consisted mainly of terrestrial resources such as C_3_ plants and terrestrial animals. Weaning trajectories varied within the population but supplements seem to have been introduced to most infants’ diets by six months of age. However, breastmilk seems to have been the most important source of protein during the first year of life. The results from this sample population suggest that between 2 and 3 years, most infants may have been completed weaned.

In summary, our data supports that in order to track individual changes in breastfeeding and weaning practices (as well as inter-individual variation in BWPs), it is preferable to select samples representing different periods of growth and development that correspond well to these stages. Although isotope proxies from both collagen (carbon and nitrogen) and dental enamel bioapatite (carbon and oxygen) possess inherent limitations and interpretive complications, they offer a better potential to describe weaning trajectories when analyzed together. This study suggests that the limits between exclusive breastfeeding and the first stage of the weaning process (start of weaning) cannot be detected precisely by analyzing either δ^18^O or δ^15^N alone. If the first stage of the weaning process is characterized by the introduction of supplements low in protein and a high nursing frequency, nitrogen isotope values would remain high. The sources of δ^15^N variability discussed before, and the lack of sensitivity to detect low protein food intake during the weaning process, make it essential to combine multiple isotope systems from different sources (e.g., collagen and bioapatite) when trying to understand the variable weaning trajectories of individuals within a population. At the same time, frequent breastmilk consumption would maintain enriched oxygen isotope values, which is further affected by the other sources of δ^18^O variation discussed above, especially the preparation of food supplements with boiled water. In this respect, δ^13^C_en_ values showed potential to detect the gradual transition from exclusive breastfeeding to complete weaned children. The third stage of the breastfeeding and weaning process (c-d) is likely signaled by a depletion of both δ^15^N and δ^13^C_co_ because of a higher protein intake and a progressive reduction of suckling. However, it is important to use models that incorporate considerations of collagen turnover rates, such as the WARN model, to overcome signal delays. According to our data, the end of the weaning process is reflected in both δ^18^O and δ^13^C_en_ values, especially when weaning foods are isotopically different from adult food. As such, the combination of multiple isotope analyses of multiple tissues formed at different ages offers the potential to overcome some of the limitations posed by single tissue approaches. It should be noted, however, that while the inclusion of deciduous teeth offers clear advantages in terms of proving new insights into various aspects of BWPs, including inter-individual (and age-related) variation, this sampling strategy is by definition limited to non-adults and as a consequence to non-survivors in the broadest sense. A future study, using high resolution microscopy in tooth enamel and incremental dentine layers along with analysis of rib and femur bone collagen of the same individuals will help to refine some of the conclusions reached in this study.
